# Catatonia and multiple pressure ulcers: A rare complication in psychiatric setting

**DOI:** 10.4103/0019-5545.55091

**Published:** 2009

**Authors:** Abhishek Srivastava, Anupam Gupta, Pratima Murthy, Thyloth Murali

**Affiliations:** Department of Psychiatric and Neurological Rehabilitation, National Institute of Mental Health and Neurosciences, Bangalore, India; 1 Department of Psychiatry, National Institute of Mental Health and Neurosciences, Bangalore, India

**Keywords:** Catatonia, depression, healing, pressure ulcer, multidisciplinary team

## Abstract

The incidence of pressure ulcers in patients with psychiatric illness, especially with catatonia might be more than what is reported in the literature. We report a case of catatonia secondary to severe depression presenting with multiple pressure ulcers. Single case report — description and management. An 18 yrs old boy reported with a continuous course illness characterized by features of catatonia secondary to severe depression with multiple pressure ulcers over sacrum and heels. Ulcers were effectively managed by a multidisciplinary team of physiatrist, psychiatrist, and rehabilitation nurses. Immobility, reduced nocturnal movements, increased skin fragility, and poor nutrition contribute to the development of the pressure ulcer in bed-bound psychiatric patients. Efforts should be directed toward the prevention of pressure ulcers in these patients to reduce additional morbidity.

## INTRODUCTION

Pressure ulcer is best described as “an area of unrelieved pressure over a defined area,” usually over a bony prominence, resulting in ischemia, cell death, and tissue necrosis.[1] The incidence of pressure ulcer in hospitalized patients ranges from 2.7% to 29% and prevalence from 3.5% to 69%.[2] The prevalence of pressure ulcers in psychiatric hospitals ranges from 1.4% to 3.8% in older people above 65 years and 0.0-0.8% in younger patients.[3] The common causes for the development of pressure ulcer in patients with psychiatric diagnosis are: impaired consciousness, dementia, Parkinson's disease,[4] depression,[5] altered psychological behavior, or splint usage in psychotic patients.[6] A common factor in all these conditions is immobility, leading to prolonged unrelieved pressure, tissue ischemia, and cell death. Pressure ulcers increase length of stay, escalate the cost of treatment, and impair quality of life. We report a case with multiple pressure ulcers secondary to catatonia in a patient with severe depression and how he was managed by combined efforts of a multidisciplinary team.

## CASE REPORT

**Figure 1:**
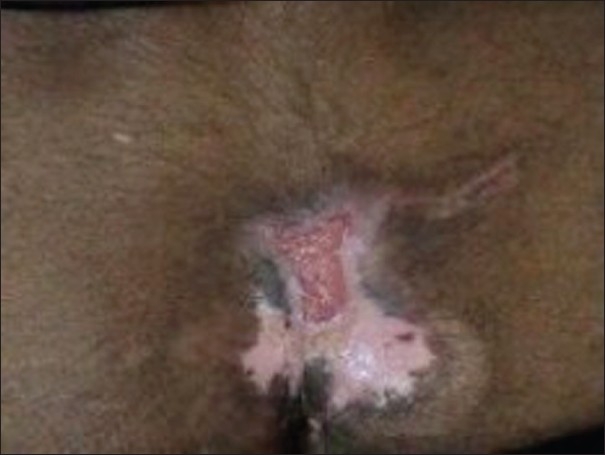
Sacral pressure ulcer

**Figure 2:**
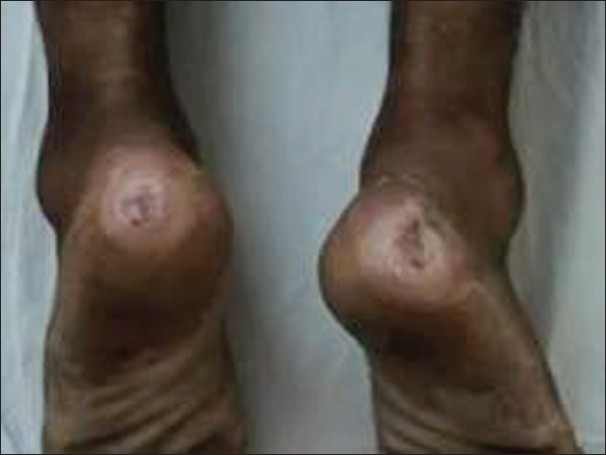
Bilateral heel ulcers

An 18 yrs old man with no significant past, personal, or family history with well-adjusted pre-morbid personality presented with an acute-onset continuous illness characterized by low mood and withdrawn behavior for last 9 months, mutism, staring and stereotypic behavior with posturing, reduced oral intake, and negativism for 3 months and multiple ulcers over back and heels for last 1 month. The symptoms started while he was preparing for his board exams and worsened when he was unable to perform well in the examinations. At evaluation he was apathetic and had open eyes with fixed gaze but no emotional responsiveness and reaction to stimuli. He was mute with no spontaneous acts and no rigidity. He was poorly kempt and had asthenic built. Systemic examination was normal. He had three pressure ulcers (details follow). He was diagnosed as a case of catatonia with severe depression and multiple pressure ulcers. Routine hemogram and biochemistry were within normal limits. He received oral benzodiapines (Tab. lorazepam 2 mg 1-1-1), antibiotics (Cap. amoxycillin 500 mg 1-1-1), and vitamin supplements. He was referred to the department of psychiatric and neurological rehabilitation for the management of pressure ulcers. He had three pressure ulcers-one rectangular shaped, grade III ulcer over sacral region 5.0 × 2.5 cm in size, clean and granulating [Figure 1], and two circular ulcers over bilateral heel grade II, 1 cm in diameter with necrotic slough [Figure 2]. Wound swab cultures showed growth of Enterobacter species sensitive to amoxicillin. There was no evidence of osteomyelitis on-rays of pelvis and ankle and foot. He was put on conservative regimen of management consisting of nursing on water mattress, proper bed positioning, regular pressure relief, daily debridement and dressing of pressure ulcers, and appropriate antibiotic as per the culture along with the continuation of the supportive medications. Catatonic features responded initially to benzodiapines, but later electroconvulsive therapy was initiated and seven sessions were conducted. The patient responded well to the combined approach and was shifted to oral anti-depressant (Cap. fluoxetine 20 mg 1-0-0) and was discharged after an in-patient stay of over 8 weeks. At the time of discharge, he was euthymic with adequate oral intake, taking medications regularly, and all pressure ulcers were completely healed.

## DISCUSSION

Pressure ulcers are caused by the interaction of multiple, diverse, etio-pathological factors that can be classified as patho-mechanical or patho-physiological.[7] Common patho-mechanical (extrinsic or primary) factors are prolonged pressure and immobility along with shear and friction, whereas fever, anemia, malnutrition, decreased lean body mass, and neurological disease are common patho- physiological (intrinsic or secondary) factors.

Immobility in bed tends to cause pressure ulcers on occiput, sacrum, heels, malleoli, and trochantric regions.[8] Our patient had ulcers over sacrum and heel. Nocturnal movements associated with sleep tends to decrease as hospital stay increases,[9] and analysis of periodic body movements in persons at risk for pressure ulcers suggests a relationship between spontaneous body movement and the development of pressure ulcer.[9] Our patient developed ulcers before admission to the hospital but must have had reduced body movement at night. There is increased association of skin fragility and poor healing with an altered psychological behavior. This combination of vulnerability to recurrent pressure sores in association with the pathological intellectual debility is described as ectodermic syndrome[6] that might have also contributed to ulcer generation in our patient. Our patient had lean body mass (BMI: 19, low normal) but hemoglobin (12.6 g/dl), serum albumin (3.9 g/dl), and absolute lymphocyte count (2240/mm^3^) were within normal range suggesting minimal contribution from patho-physiological factors in ulcer development in spite of reduced oral intake for last few weeks.

It is imperative to treat the patient's medical condition that predisposes to pressure ulcers. If possible, the patho-physiological factors should be controlled in conjunction with the elimination of the patho-mechanical factors. This is possible by combined efforts of a multidisciplinary team. In our case, medications and electroconvulsive therapy were started to eliminate the primary pathology by the psychiatry team. The rehabilitation team provided proper care of the ulcers by conservative regimen consisting of appropriate medications, dressings, special mattress, advice for proper nursing care, and early mobilization for controlling the physiological factors and early healing of the ulcers.

## CONCLUSION

The incidence of pressure ulcers in patients with psychiatric illness, especially with catatonia might be more than what is reported in the literature. Pressure ulcers significantly increase length of stay, morbidity, and the cost of management. All efforts should be directed toward the prevention of pressure ulcers in bed-bound psychiatric patients to reduce additional morbidity. Pressure ulcers in patients with psychiatric illness can be managed effectively by combined and coordinated efforts of a multidisciplinary team.
